# Supplementary material for: ARF suppression by MYC but not MYCN confers increased malignancy of aggressive pediatric brain tumors
Source: Nat Commun. 2023 Mar 3;14:1221. doi: 10.1038/s41467-023-36847-9 (PMC9984535; doi:10.1038/s41467-023-36847-9)
Supplement: Supplementary file 1 — Supplementary Information [file 41467_2023_36847_MOESM1_ESM.pdf]

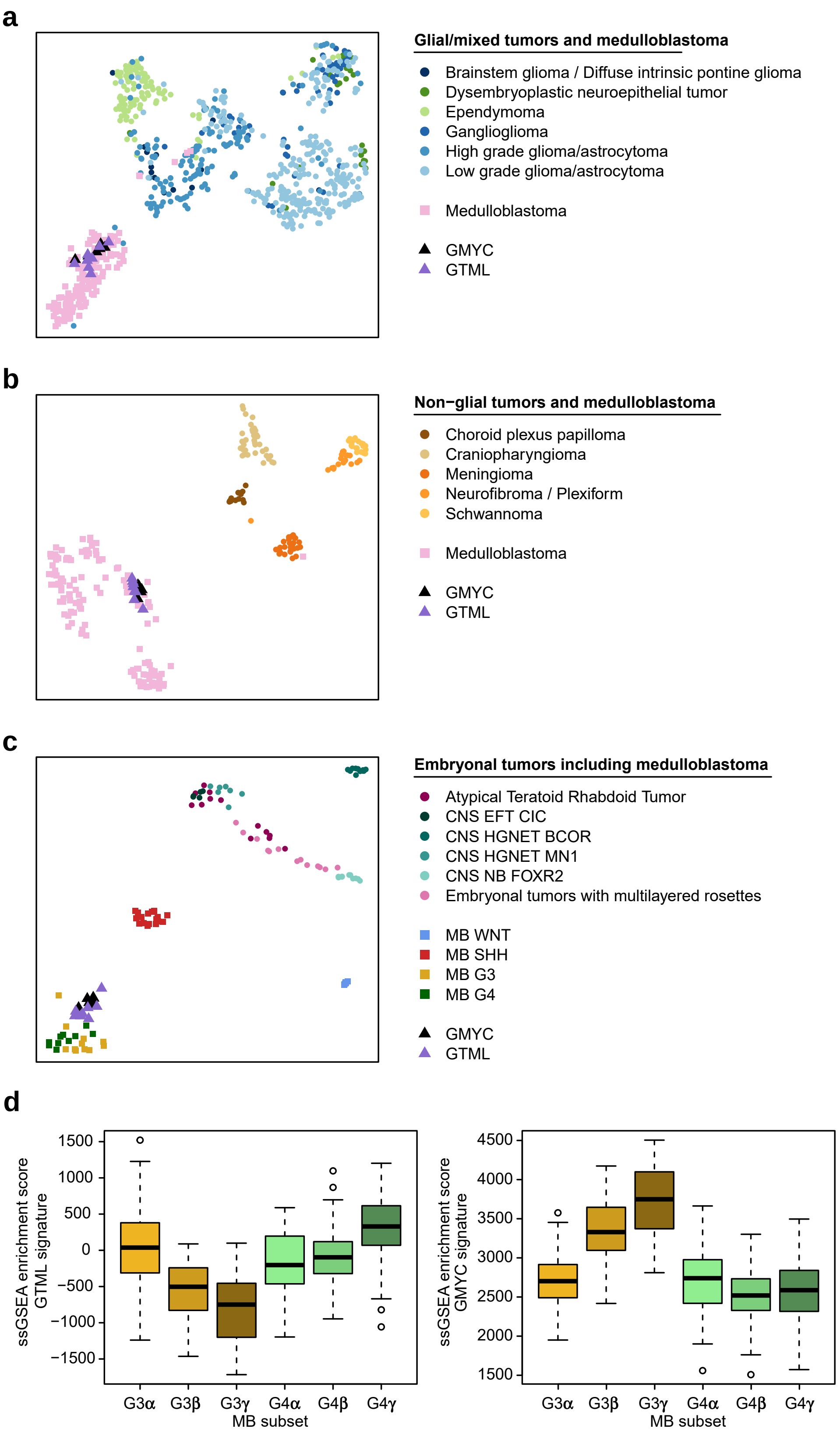

Supplementary Fig. 1. Cross-species comparison to pediatric tumor data sets demonstrates that GMYC and GTML resemble human MB.

a. t-SNE plot comparing GMYC and GTML tumor samples (colored triangles) to various human glial and mixed brain tumors (colored circles) and MB (squares; human tumor data from CBTTC database).

b. t-SNE plot comparing GMYC and GTML tumor samples (colored triangles) to various human non-glial brain tumors (colored circles) and MB (squares; human tumor data from CBTTC database).

c. t-SNE plot comparing GMYC and GTML tumor samples (colored triangles) to various embryonal brain tumors (colored circles), including MB (squares of different subtypes; human tumor data from GSE73038). All t-SNE plots were produced from the metagene expression values obtained after cross-species mapping of transcriptional profiles from mouse tumors onto human tumors.

d. ssGSEA enrichment analysis of GMYC and GTML tumors against human MB subtypes (GSE85217). GTML tumours appear to best resemble G3α and G4-like MB and GMYC tumors appear to best resemble G3γ-like MB.

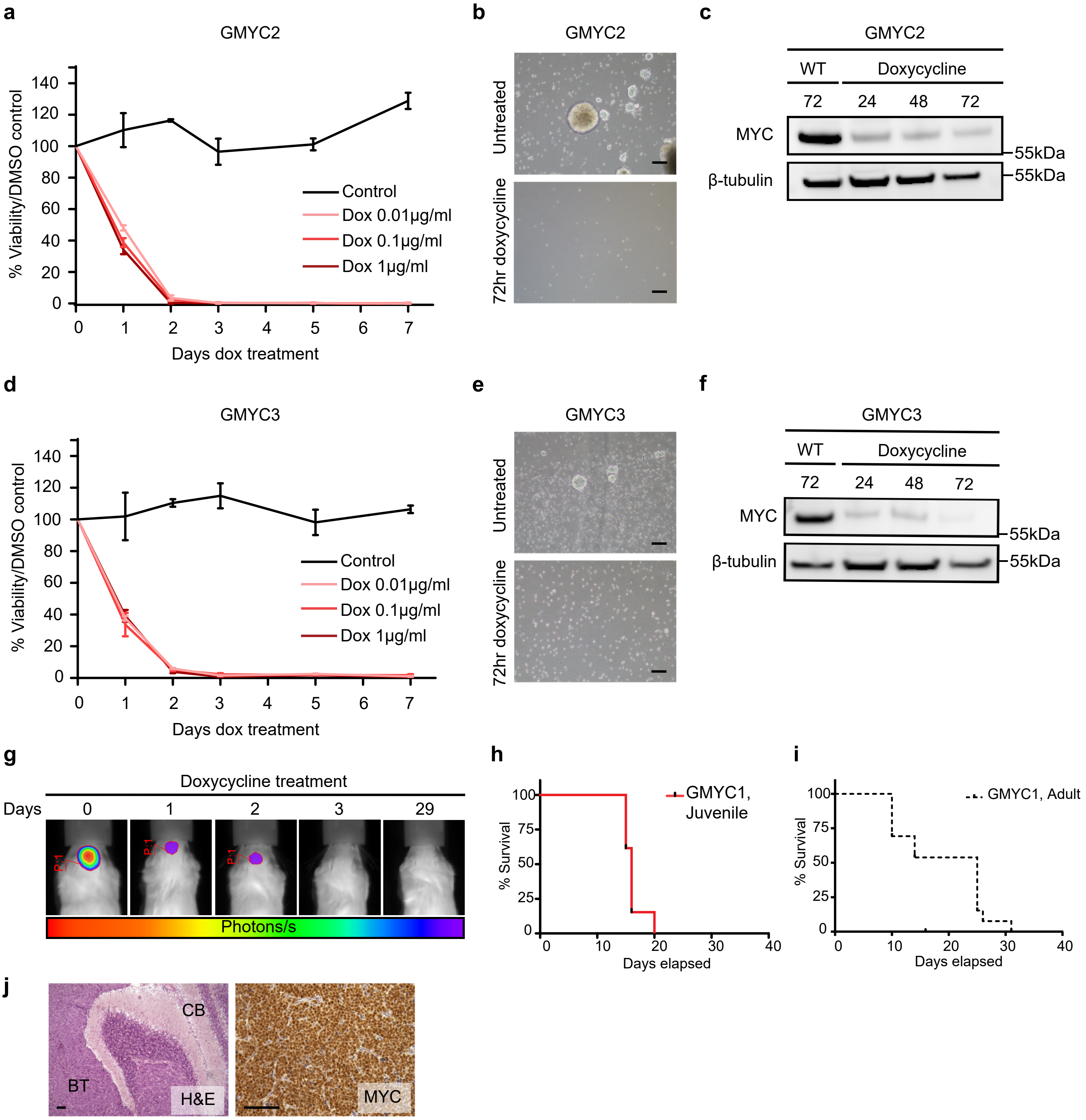

Supplementary Fig. 2. MYC suppression leads to complete ablation of tumor cells and survival of mice. GMYC tumors likely arise during embryonal development and can be maintained in vitro.

a. GMYC2 cells can be dox treated *in vitro* and are rapidly ablated following exposure. Mean ± SD.

b. Micrograph of GMYC2 cells growing *in vitro*, both untreated and treated for 72 hours with dox. Scale bars represent 100µM.

c. Protein analysis of dox treated GMYC2 cells *in vitro* over 72 hours.

d. GMYC3 cells can be dox treated in vitro and are rapidly ablated following exposure. Mean ± SD.

e. Micrograph of GMYC3 cells growing in vitro, both untreated and treated for 72 hours with dox. Scale bars represent 100µM.

f. Protein analysis of dox treated GMYC3 cells *in vitro*.

g. A GMYC/TreCRE-LC1 mouse followed during dox treatment over approx. a month. Day 0 indicates when the tumor was phenotypically visible on the mouse. Dox-mediated *MYC* suppression rapidly leads to complete clearance of Luc-positive tumor cells.

h. Survival plot of juvenile immunocompetent FVBN mice who received cerebellar injections of 200,000 GMYC1 cells.

i. Survival plot of adult FVBN mice who received cerebellar injections of 100,000 GMYC1 cells.

j. H&E and MYC immunostaining of allografted tumors show cerebellar disruption and high levels of MYC protein. Scale bars represent 100µM.

All experimental data from immunostainings and treatments (**a-g**, **j**) was verified from at least two independent biological replicates.

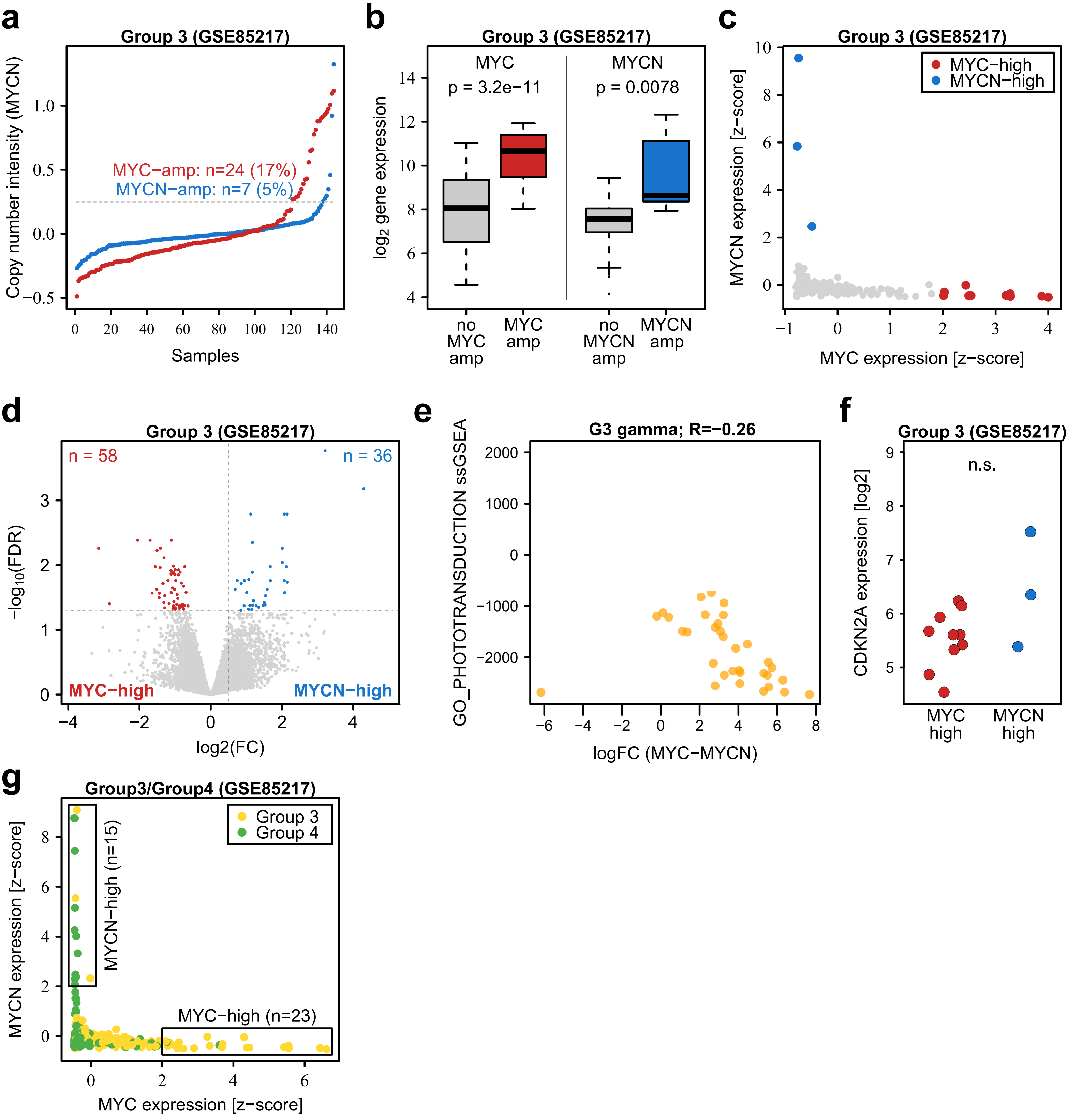

Supplementary Fig. 3. Identification and comparison of MYC-high/amplified and MYCN-high/amplified Group 3 MBs.

a. Illustration of the estimation of putative copy number events of *MYC* (red) and *MYCN* (blue) in Group 3 MBs based on methylation-derived copy number intensity values. Dashed line indicates the cut off (>0.25) for selecting samples with putative amplification. Numbers above the dashed line represent the identified number of amplified cases for *MYC* (red) and *MYCN* (blue).

b. Boxplots comparing the gene expression of *MYCN* or *MYC* between samples with putative amplifications in that gene and samples without the amplification. P-values were computed using one-sided Welch's t-test.

c. Scatter plot comparing the z-score of gene expression values (in normal scale) between *MYC* and *MYCN* within Group 3 MBs. MYC-high and MYCN-high samples were selected as those cases with z>=2 for the respective gene.

d. Volcano plot depicting the differential expression results between the MYC-high (n=10) and MYCN-high (n=3) Group 3 MBs. *MYC* and *MYCN* were removed from the expression data prior to the differential analysis. The horizontal dashed line indicates the FDR=0.05 threshold, while the vertical dashed lines indicate a logFC of -0.5 or 0.5, respectively.

e. Scatter plot comparing the ssGSEA enrichment score for a phototransduction gene set (identified in Fig. 4B) with the corresponding *MYC* expression levels in human Group 3-gamma patients. The R-value reflects the Pearson correlation coefficient.

f. Strip chart comparing the gene expression of *CDKN2A* between MYC-high (n=10) and MYCN-high (n=3) Group 3 MBs. The p-value (n.s.: p>0.05) was computed using a two-sided Welch's t-test.

g. Scatter plot comparing the z-score of gene expression values (in normal scale) between *MYC* and *MYCN* within Group 3 and Group 4 MBs. MYC-high and MYCN-high samples were selected as those cases with z>=2 for the respective gene.

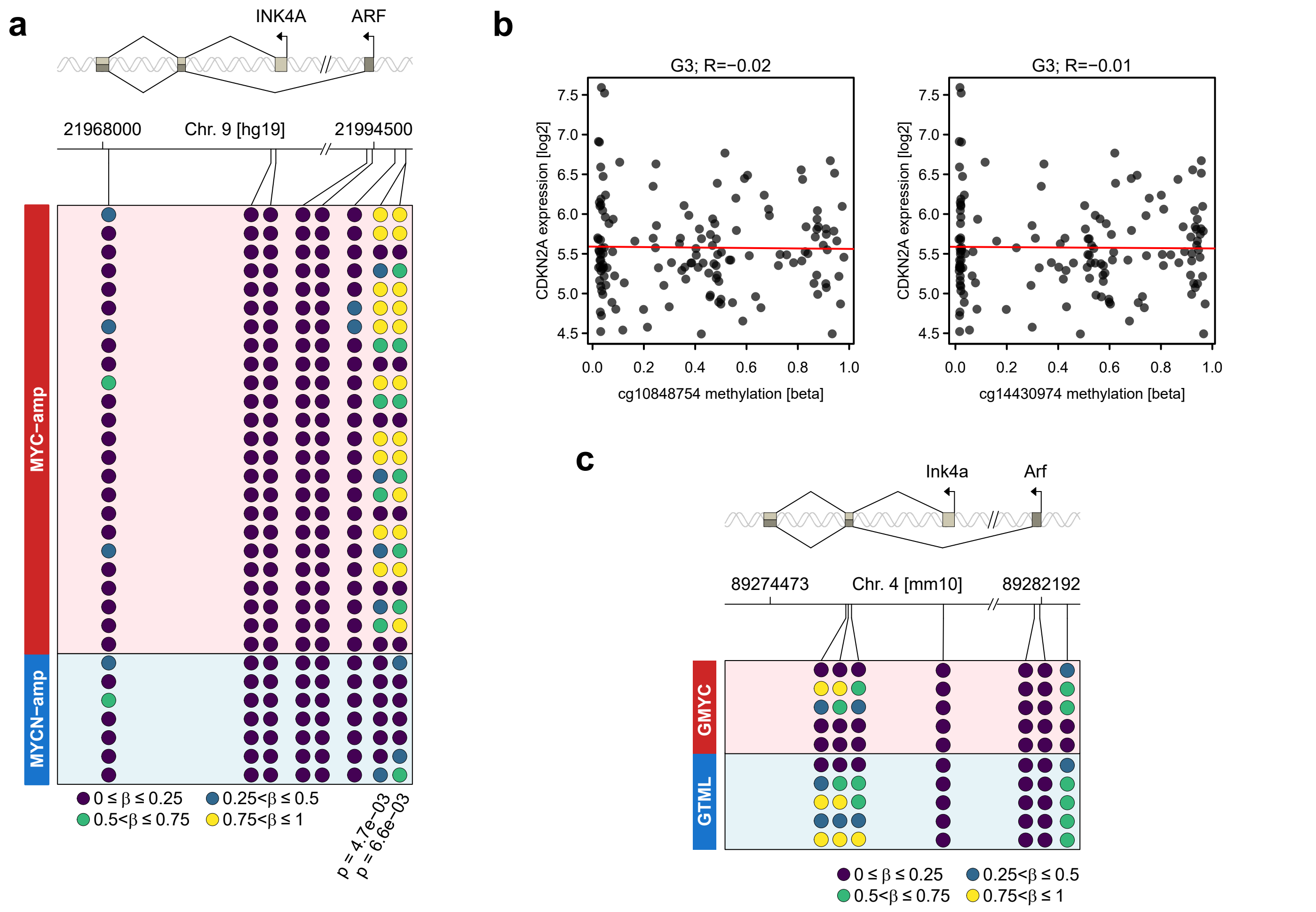

Supplementary Fig. 4. Methylation analysis of CDKN2A locus in human Gr. 3 MB and in mouse brain tumors.
a. Comparison of *CDKN2A* methylation between human Group 3 samples with putative MYC amplification and Group 3 samples with putative MYCN amplification. The position of CpG probes is shown relative to the exons utilized by *ARF* and/or *INK4A.*
b. Comparison of expression levels of CDKN2A of hyper- and hypo-methylated samples from human Group 3 MB.
c. Comparison of *Cdkn2a* methylation in tumor DNA between GMYC and GTML models using MM285 Infinium Mouse Methylation BeadChip. The position of CpG probes is shown relative to the exons utilized by *Arf* and/or *Ink4a.*

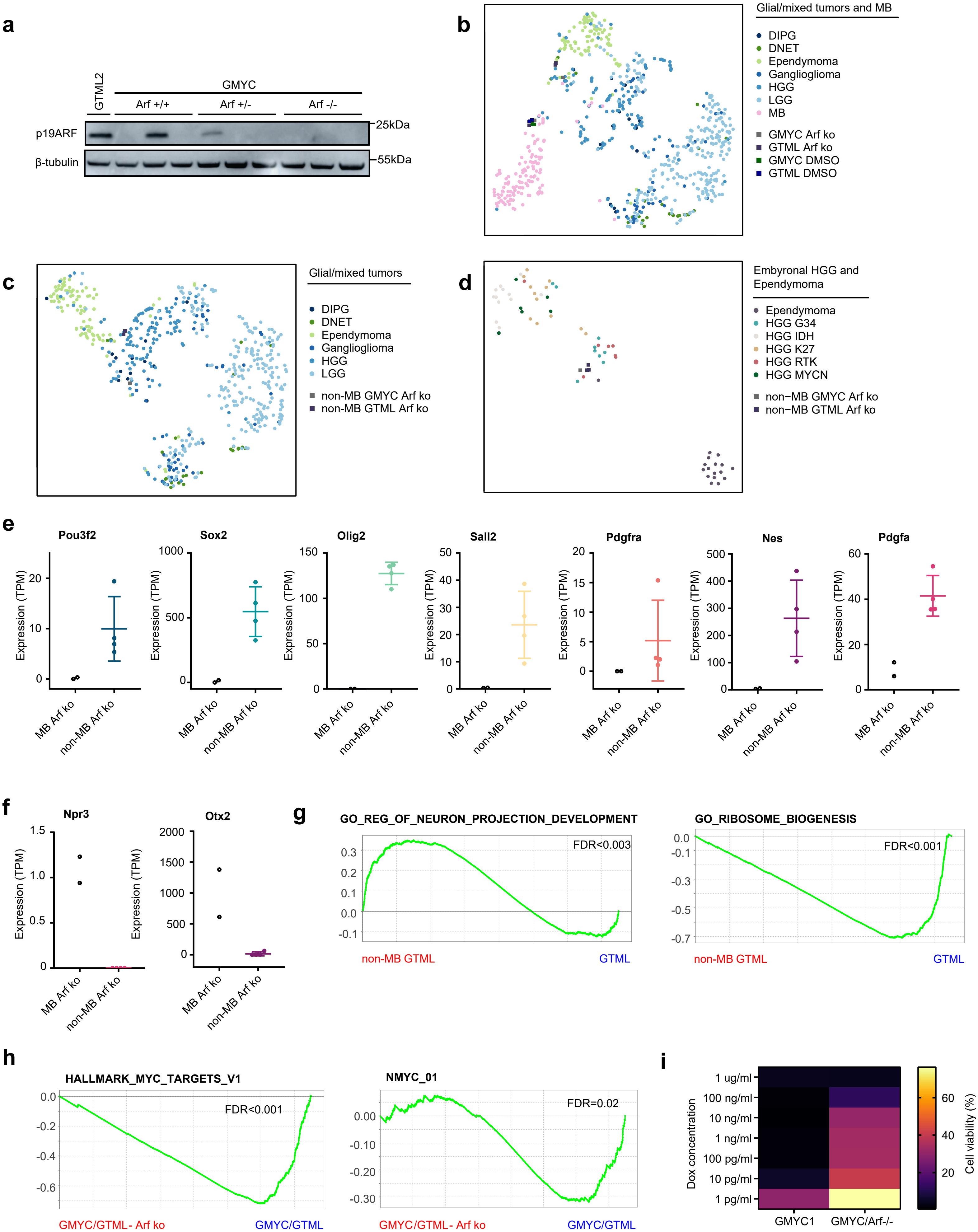

a
GTML2
GMYC
Arf +/+
Arf +/-
Arf -/-
p19ARF
β-tubulin
25kDa
55kDa
b
Glial/mixed tumors and MB
DIPG
DNET
Ependymoma
Ganglioglioma
HGG
LGG
MB
GMYC Arf ko
GTML Arf ko
GMYC DMSO
GTML DMSO
c
Glial/mixed tumors
DIPG
DNET
Ependymoma
Ganglioglioma
HGG
LGG
non-MB GMYC Arf ko
non-MB GTML Arf ko
d
Embryonal HGG and Ependymoma
Ependymoma
HGG G34
HGG IDH
HGG K27
HGG RTK
HGG MYCN
non−MB GMYC Arf ko
non−MB GTML Arf ko
e
Pou3f2
Sox2
Olig2
Sall2
Pdgfra
Nes
Pdgfa
Expression (TPM)
MB Arf ko
non-MB Arf ko
f
Npr3
Otx2
g
GO_REG_OF_NEURON_PROJECTION_DEVELOPMENT
FDR<0.003
GO_RIBOSOME_BIOGENESIS
FDR<0.001
non-MB GTML
GTML
h
HALLMARK_MYC_TARGETS_V1
FDR<0.001
NMYC_01
FDR=0.02
GMYC/GTML- Arf ko
GMYC/GTML
i
1 ug/ml
100 ng/ml
10 ng/ml
1 ng/ml
100 pg/ml
10 pg/ml
1 pg/ml
Dox concentration
GMYC1
GMYC/Arf-/-
Cell viability (%)

Supplementary Fig. 5. ARF depleted GMYC/GTML animals promote an increased glioma-like tumor development.
a. Protein analysis of p19ARF in the GTML2 cell line and fresh tumor biopsies taken from GMYC mice that were wildtype for *Cdkn2a/p19Arf* or had partial or complete knockout of the *Arf* gene.
b-d. tSNE plots for the *Arf* ko GMYC and GTML tumours (colored squares) that appear to be either MB-like resembling human MB (pink) or non-MB-like and then more closely similar to human HGG-G34 or HGG-RTKs when performing cross-species RNA seq analysis. The same human data sets from Supplementary Fig. 1 were used here.
e. Transcriptional profiling expression patterns for markers defining glioma propagation transcription factors (Pou3f2, Sall2, Sox2, Olig2) and upregulation of Pdgfra, Nes and Pdgfa commonly involved in HGG-RTKs. MB Arf ko (n = 2). Non-MB Arf ko (n = 4). Scatter dot plot presented as mean values +/- SD for non-MB Arf ko.
f. Transcriptional profiling expression patterns for Group 3 MB-specific markers (Npr3 and Otx2) lost in HGGs. MB Arf ko (n = 2). Non-MB Arf ko (n = 4). Scatter dot plot presented as mean values +/- SD for non-MB Arf ko.
g. Significant gene set enrichment in regulation of neuron projection development (GO_REGULATION_OF_NEURON PROJECTION_DEVELOPMENT) in non-MB GTML tumors as compared to MB GTML tumors in were ribosome biogenesis (GO_RIBOSOME_BIOGENESIS) instead was significantly enriched.
h. Significant gene set enrichment of MYC hallmark gene sets (HALLMARK_MYC_TARGETS_V1) and MYCN target gene sets (NMYC_01.v7.5.1 from GSE107405) in Arf wild type as compared to Arf knock out GTML and GMYC tumor lines.
i. Heat map showing cell viability of GMYC1 and GMYC Arf ko cells based on Alamar Blue assay after 72 h dox treatment at various concentrations as indicated. A significant difference ($p<0.05$) in response was found between the lines when using a two-tailed Student´s t-test.
All experimental data from immunostainings (**a**) or treatments (**i**) was verified from at least two independent biological replicates.

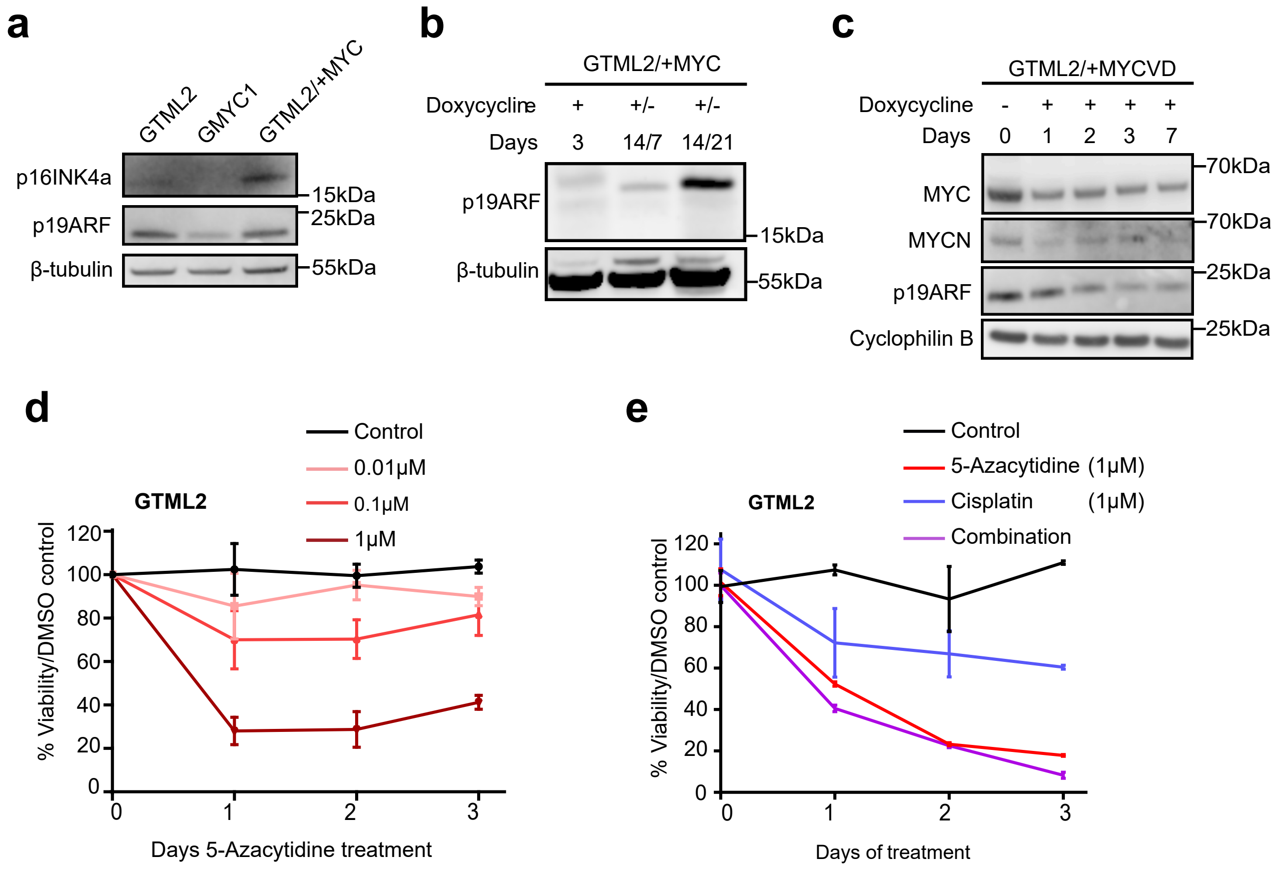

Supplementary Fig. 6. MYC is suppressing ARF levels and MYCN-driven GTML2 cells treated with 5-Azacytidine show a decrease in cell survival.

a. Protein analysis of p16INK4a and p19ARF in untreated GTML2, GMYC1, and GTML2/+MYC cell lines.

b. Protein analysis of GTML2/+MYC cell line during dox treatment. 14/7 and 14/21 indicate days of dox treatment and days released from treatment. MYC promotes temporary de novo methylation of the *ARF* gene, indicated with less protein. Removal from dox, and hence restoration of the original MYCN driver, leads to restoration of the *ARF* gene and its product.

c. GTML2/+MYCVD cells treated with dox in vitro over a period of 7 days show stable levels of MYC, a decrease of MYCN levels during dox treatment, and reduction of total ARF protein.

d. GTML2 cells were treated in vitro with 5-Azacytidine over 3 days. The highest concentration (1μmol/L) caused a reduction in cell viability and proliferation. n = 3 for each treatment variable. Mean ± SD.

e. GTML2 cells were then treated in vitro with 5-Azacytidine (demethylation), cisplatin (alkylation), or combination treatment over 3 days. Independent treatments saw a reduction in cell viability and proliferation. n = 3 for each treatment variable. Mean ± SD. All experimental data from immunostainings (**a-c**) and treatments (**d-e**) was verified from at least 2 biological replicates.

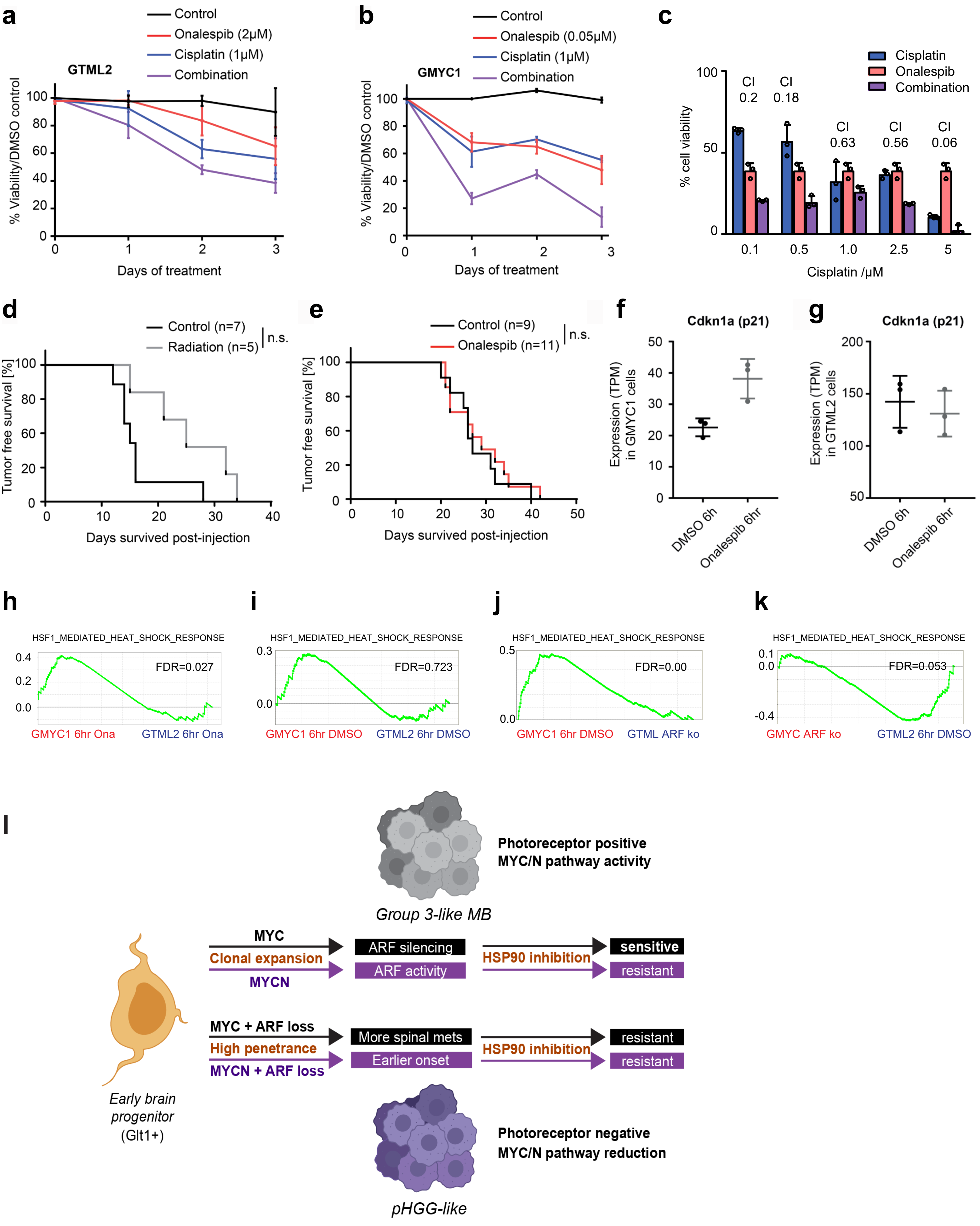
a
GTML2
Control
Onalespib (2μM)
Cisplatin (1μM)
Combination
% Viability/DMSO control
Days of treatment
b
GMYC1
Control
Onalespib (0.05μM)
Cisplatin (1μM)
Combination
% Viability/DMSO control
Days of treatment
c
Cisplatin
Onalespib
Combination
% cell viability
CI 0.2
CI 0.18
CI 0.63
CI 0.56
CI 0.06
Cisplatin /μM
d
Control (n=7)
Radiation (n=5)
n.s.
Tumor free survival [%]
Days survived post-injection
e
Control (n=9)
Onalespib (n=11)
n.s.
Tumor free survival [%]
Days survived post-injection
f
Cdkn1a (p21)
Expression (TPM) in GMYC1 cells
DMSO 6h
Onalespib 6hr
g
Cdkn1a (p21)
Expression (TPM) in GTML2 cells
DMSO 6h
Onalespib 6hr
h
HSF1_MEDIATED_HEAT_SHOCK_RESPONSE
FDR=0.027
GMYC1 6hr Ona
GTML2 6hr Ona
i
HSF1_MEDIATED_HEAT_SHOCK_RESPONSE
FDR=0.723
GMYC1 6hr DMSO
GTML2 6hr DMSO
j
HSF1_MEDIATED_HEAT_SHOCK_RESPONSE
FDR=0.00
GMYC1 6hr DMSO
GTML ARF ko
k
HSF1_MEDIATED_HEAT_SHOCK_RESPONSE
FDR=0.053
GMYC ARF ko
GTML2 6hr DMSO
l
Photoreceptor positive
MYC/N pathway activity
Group 3-like MB
MYC
Clonal expansion
MYCN
ARF silencing
ARF activity
HSP90 inhibition
sensitive
resistant
MYC + ARF loss
High penetrance
MYCN + ARF loss
More spinal mets
Earlier onset
HSP90 inhibition
resistant
resistant
Early brain
progenitor
(Glt1+)
Photoreceptor negative
MYC/N pathway reduction
pHGG-like

Supplementary Fig. 7. MYC-driven MB-like tumors are more sensitive to HSP90 inhibition as compared to MYCN-driven MB-like tumors.
a. GTML2 cells treated with Onalespib, cisplatin or combination treatment over 3 days. Independent treatments reduced cell viability. Combinatorial treatment was non-significant nor synergistic n = 3 for each treatment variable. Mean ± SD.
b. GMYC1 cells treated with Onalespib, cisplatin, or combination treatment over 3 days. Independent treatments reduced cell viability. n = 3 for each treatment variable. Mean ± SD.
c. Synergism scores and combinatorial index (CI) from GMYC1 cells treated with Onalespib (0.1µM) and varying conc. of cisplatin over 3 days. The combinatorial treatment had a synergistic effect on these cells. n = 3 for each treatment variable. Mean ± SD.
d. GMYC/TetGFP/Luc cells injected into the cerebellum of immunocompetent mice, which underwent a daily regimen of 2 Gy irradiation for 5 days or received I.P. injections of vehicle (2-hydroxypropyl-β-cyclodextrin). Irradiation increased overall survival but was not significant (p=0.0536). Log-rank Mantel-Cox statistical test.
e. GMYC/ARFKO cells injected into the cerebellum of immunocompetent mice, which underwent daily I.P. injections of 20 mg/kg of Onalespib or vehicle (2-hydroxypropy-β-cyclodextrin) for 4 days. Onalespib treatment did not significantly increase survival (p=0.7857). Log-rank Mantel-Cox statistical test.
f-g. RNA expression data of GMYC1 and GTML2 cells treated with high-dose Onalespib over 6 hours. *p21* expression increased in GMYC1 cells as compared to GTML2 cells, indicative of HSP90 inhibitory treatment and subsequent effects on MDM2 pathway. DMSO 6hr n = 3. Onalespib 6hr n = 3. Scatter dot plot presented as mean values +/- SD.
h-k. GSEA analysis of GMYC1 and GTML2 cells (treated with Onalespib, DMSO or with Arf ko) with enrichment of the REACTOME_REGULATION_OF_HSF1_MEDIATED_HEAT_SHOCK_RESPONSE gene set. Enrichments were considered significant if FDR < 0.05.
l. Overview of the distinct tumor development from Glt1-positive brain cells in this model system. While MYC and MYCN both generate photoreceptor-positive brain tumors, MYC generates MB-like tumors that are sensitive to HSP90 inhibition. MYC-driven tumors show significantly lower ARF expression compared to MYCN-driven tumors. MYC or MYCN in combination with ARF depletion promotes formation of photoreceptor-negative pediatric HGG (pHGG)-like tumors that show less prominent MYC pathway dependency and less sensitivity to HSP90 inhibitors. Figure was partly created with BioRender.com.
All experimental data from treatments (**a**-**c**) was verified in at least two independent biological replicates.

Supplementary Table 1. Trp53 mutations found after sequencing of 24 GMYC and 12 GTML tumor biopsies.

Sequencing results for evaluation of mutational status of *Trp53* in GMYC and GTML tumors. .

Sample	Model	#CHROM	POS	REF	ALT	Annotation	Annotation Impact	Variant Multplicity	Gene Name	Gene_ID	Feature_ID	Transcript BioType	Rank	HGVS.c	HGVS.p	cDNA.pos /length	CDS.pos /length	AA.pos /length	Tot. depth	Variant depth	Allele freq
Sample-5_S5	GMYC	chr11	69589629	G	C	missense_variant	MODERATE	1	Trp53	ENSMUSG00000059552	ENSMUST00000108658.9	protein_coding	8/11	c.830G>C	p.Arg277Thr	987/1771	830/1173	277/390	167	49	29,34%
Sample-9_S9	GMYC	chr11	69588362	A	T	splice_acceptor_variant & intron_variant	HIGH	1	Trp53	ENSMUSG00000059552	ENSMUST00000108658.9	protein_coding	4/10	c.367-2A>T					107	90	84,11%
Sample-19_S3	GMYC	chr11	69588505	G	A	missense_variant	MODERATE	1	Trp53	ENSMUSG00000059552	ENSMUST00000108658.9	protein_coding	5/11	c.508G>A	p.Val170Met	665/1771	508/1173	170/390	177	87	49,15%
Sample-21_S5	GMYC	chr11	69588461	G	C	missense_variant	MODERATE	3	Trp53	ENSMUSG00000059552	ENSMUST00000108658.9	protein_coding	5/11	c.464G>C	p.Arg155Pro	621/1771	464/1173	155/390	163	107	65,64%
Sample-24_S8	GMYC	chr11	69589607	C	T	missense_variant	MODERATE	1	Trp53	ENSMUSG00000059552	ENSMUST00000108658.9	protein_coding	8/11	c.808C>T	p.Arg270Cys	965/1771	808/1173	270/390	184	152	82,61%
Sample-25_S9	GMYC	chr11	69588393	C	G	missense_variant	MODERATE	3	Trp53	ENSMUSG00000059552	ENSMUST00000108658.9	protein_coding	5/11	c.396C>G	p.Cys132Trp	553/1771	396/1173	132/390	114	40	35,09%
Sample-26_S10	GMYC	chr11	69589608	G	T	missense_variant	MODERATE	1	Trp53	ENSMUSG00000059552	ENSMUST00000108658.9	protein_coding	8/11	c.809G>T	p.Arg270Leu	966/1771	809/1173	270/390	197	92	46,70%
Sample-27_S11	GMYC	chr11	69589608	G	A	missense_variant	MODERATE	1	Trp53	ENSMUSG00000059552	ENSMUST00000108658.9	protein_coding	8/11	c.809G>A	p.Arg270His	966/1771	809/1173	270/390	148	89	60,14%
Sample-27_S11	GMYC	chr11	69589635	G	A	missense_variant	MODERATE	1	Trp53	ENSMUSG00000059552	ENSMUST00000108658.9	protein_coding	8/11	c.836G>A	p.Arg279His	993/1771	836/1173	279/390	164	54	32,93%
Sample-28_S12	GMYC	chr11	69588382	A	G	missense_variant	MODERATE	1	Trp53	ENSMUSG00000059552	ENSMUST00000108658.9	protein_coding	5/11	c.385A>G	p.Lys129Glu	542/1771	385/1173	129/390	106	94	88,68%
Sample-31_S15	GMYC	chr11	69589210	C	T	missense_variant	MODERATE	1	Trp53	ENSMUSG00000059552	ENSMUST00000108658.9	protein_coding	7/11	c.733C>T	p.Arg245Cys	890/1771	733/1173	245/390	129	109	84,50%
Sample-37_S21	GTML	chr11	69588461	G	C	missense_variant	MODERATE	3	Trp53	ENSMUSG00000059552	ENSMUST00000108658.9	protein_coding	5/11	c.464G>C	p.Arg155Pro	621/1771	464/1173	155/390	188	96	51,06%
Sample-40_S24	GTML	chr11	69588512	G	A	missense_variant	MODERATE	2	Trp53	ENSMUSG00000059552	ENSMUST00000108658.9	protein_coding	5/11	c.515G>A	p.Arg172His	672/1771	515/1173	172/390	168	138	82,14%
Sample-40_S24	GTML	chr11	69588393	C	G	missense_variant	MODERATE	3	Trp53	ENSMUSG00000059552	ENSMUST00000108658.9	protein_coding	5/11	c.396C>G	p.Cys132Trp	553/1771	396/1173	132/390	144	17	11,81%
Sample-42_S26	GTML	chr11	69588461	G	C	missense_variant	MODERATE	3	Trp53	ENSMUSG00000059552	ENSMUST00000108658.9	protein_coding	5/11	c.464G>C	p.Arg155Pro	621/1771	464/1173	155/390	210	86	40,95%
Sample-44_S28	GTML	chr11	69588463	G	C	missense_variant	MODERATE	1	Trp53	ENSMUSG00000059552	ENSMUST00000108658.9	protein_coding	5/11	c.466G>C	p.Ala156Pro	623/1771	466/1173	156/390	159	88	55,35%
Sample-44_S28	GTML	chr11	69588393	C	G	missense_variant	MODERATE	3	Trp53	ENSMUSG00000059552	ENSMUST00000108658.9	protein_coding	5/11	c.396C>G	p.Cys132Trp	553/1771	396/1173	132/390	106	33	31,13%
Sample-47_S31	GTML	chr11	69588512	G	A	missense_variant	MODERATE	2	Trp53	ENSMUSG00000059552	ENSMUST00000108658.9	protein_coding	5/11	c.515G>A	p.Arg172His	672/1771	515/1173	172/390	121	32	26,45%

Supplementary Table 2. Gene set enrichment analysis in GMYC1 and GTML2 cells after HSP90 inhibition

Significantly enriched in GMYC1 cells after 6h Onalespib treatment*	**NES**	**FDR q-val****	**SIZE**
REACTOME_COMPLEMENT_CASCADE	2,2338	0,0000	20
KRIGE_AMINO_ACID_DEPRIVATION	2,1623	0,0023	22
CONCANNON_APOPTOSIS_BY_EPOXOMICIN_UP	2,1341	0,0023	207
ZHAN_MULTIPLE_MYELOMA_CD1_UP	2,1258	0,0018	39
SU_KIDNEY	2,1201	0,0014	15
HELLER_SILENCED_BY_METHYLATION_DN	2,1147	0,0016	85
NIELSEN_SYNOVIAL_SARCOMA_DN	2,0916	0,0027	17
KUROZUMI_RESPONSE_TO_ONCOCYTIC_VIRUS	2,0901	0,0025	40
DELPUECH_FOXO3_TARGETS_DN	2,0864	0,0027	32
VANASSE_BCL2_TARGETS_UP	2,0830	0,0027	30
DAUER_STAT3_TARGETS_DN	2,0487	0,0047	37
GO_POSITIVE_REGULATION_OF_INTERLEUKIN_8_PRODUCTION	2,0406	0,0048	41
KEGG_COMPLEMENT_AND_COAGULATION_CASCADES	2,0287	0,0057	56
GO_REGULATION_OF_INTERLEUKIN_8_PRODUCTION	2,0181	0,0065	55
WANG_NEOPLASTIC_TRANSFORMATION_BY_CCND1_MYC	2,0121	0,0067	20
GAUSSMANN_MLL_AF4_FUSION_TARGETS_F_DN	2,0083	0,0069	30
WENG_POR_TARGETS_LIVER_DN	1,9849	0,0097	19
GO_MONOCYTE_CHEMOTAXIS	1,9845	0,0094	24
GO_NEUTRAL_AMINO_ACID_TRANSPORT	1,9823	0,0099	33
GO_CELLULAR_RESPONSE_TO_HEAT	1,9763	0,0102	30
YORDY_RECIPROCAL_REGULATION_BY_ETS1_AND_SP100_DN	1,9532	0,0120	63
GO_L_AMINO_ACID_TRANSPORT	1,9451	0,0138	56
GO_NEURONAL_ACTION_POTENTIAL	1,9374	0,0149	27
GO_CALCIUM_ION_IMPORT	1,9275	0,0164	60
GO_MULTICELLULAR_ORGANISMAL_SIGNALING	1,9260	0,0165	119
REACTOME_CHEMOKINE_RECEPTORS_BIND_CHEMOKINES	1,9027	0,0228	33
MIKKELSEN_IPS_ICP_WITH_H3K4ME3_AND_H327ME3	1,9024	0,0222	106
SHIN_B_CELL_LYMPHOMA_CLUSTER_5	1,8859	0,0275	16
MENSSEN_MYC_TARGETS	1,8820	0,0282	45
MIKKELSEN_MEF_LCP_WITH_H3K27ME3	1,8755	0,0299	58
NIKOLSKY_BREAST_CANCER_1Q21_AMPLICON	1,8625	0,0352	35
REACTOME_ACTIVATION_OF_GENES_BY_ATF4	1,8594	0,0360	22
CUI_TCF21_TARGETS_DN	1,8592	0,0352	25
KEGG_AUTOIMMUNE_THYROID_DISEASE	1,8568	0,0353	25
HANN_RESISTANCE_TO_BCL2_INHIBITOR_DN	1,8546	0,0353	44
ODONNELL_TARGETS_OF_MYC_AND_TFRC_UP	1,8514	0,0364	63
REACTOME_AMINO_ACID_TRANSPORT_ACROSS_THE_PLASMA_MEMBRANE	1,8506	0,0359	29
GAUSSMANN_MLL_AF4_FUSION_TARGETS_D_UP	1,8478	0,0362	34
KEGG_INTESTINAL_IMMUNE_NETWORK_FOR_IGA_PRODUCTION	1,8404	0,0394	30
VECCHI_GASTRIC_CANCER_EARLY_DN	1,8397	0,0388	287
GO_ACTION_POTENTIAL	1,8331	0,0412	89
GO_CHAPERONE_MEDIATED_PROTEIN_FOLDING	1,8238	0,0459	44
GAJATE_RESPONSE_TO_TRABECTEDIN_UP	1,8187	0,0473	54
GO_REGULATION_OF_CELLULAR_RESPONSE_TO_HEAT	1,8158	0,0484	69
GO_MEMBRANE_DEPOLARIZATION_DURING_ACTION_POTENTIAL	1,8149	0,0480	38
HUMMERICH_MALIGNANT_SKIN_TUMOR_DN	1,8147	0,0470	15
GO_CALCIUM_ION_IMPORT_INTO_CYTOSOL	1,8134	0,0469	40
LEI_HOXC8_TARGETS_DN	1,8070	0,0499	15

Significantly enriched in GTML2 cells after 6h Onalespib treatment

ZHAN_MULTIPLE_MYELOMA_CD1_VS_CD2_UP	2,2033	0,0000	55
REACTOME_OLFACTORY_SIGNALING_PATHWAY	2,0985	0,0018	176
TIEN_INTESTINE_PROBIOTICS_24HR_DN	2,0950	0,0012	188
KEGG_ANTIGEN_PROCESSING_AND_PRESENTATION	2,0723	0,0018	44
KRIGE_AMINO_ACID_DEPRIVATION	2,0167	0,0055	22
NIELSEN_SCHWANNOMA_UP	1,9763	0,0110	16
GO_RESPONSE_TO_TOPOLOGICALLY_INCORRECT_PROTEIN	1,9567	0,0151	142
ZHAN_MULTIPLE_MYELOMA_CD1_UP	1,9479	0,0156	39
GO_ENDOPLASMIC_RETICULUM_TO_CYTOSOL_TRANSPORT	1,9162	0,0236	20
GROSS_HYPOXIA_VIA_ELK3_ONLY_UP	1,9076	0,0253	28
WU_ALZHEIMER_DISEASE_DN	1,9014	0,0258	16
PACHER_TARGETS_OF_IGF1_AND_IGF2_UP	1,8786	0,0343	33
GO_PROTEIN_REFOLDING	1,8762	0,0332	18
GO_RESPONSE_TO_PH	1,8742	0,0322	36
HALLMARK_UNFOLDED_PROTEIN_RESPONSE	1,8650	0,0355	100
SHIN_B_CELL_LYMPHOMA_CLUSTER_5	1,8637	0,0341	16
REACTOME_AMINO_ACID_TRANSPORT_ACROSS_THE_PLASMA_MEMBRANE	1,8529	0,0391	29

**Significantly enriched publically available gene sets in GMYC1 or GTML2 cells after 6 hours of Onalespib treatment from AmpliSeq RNA sequencing analysis.*
***Cut off FDR q-val<0,05.*
