# Supplementary material for: ARF suppression by MYC but not MYCN confers increased malignancy of aggressive pediatric brain tumors
Source: Nat Commun. 2023 Mar 3;14:1221. doi: 10.1038/s41467-023-36847-9 (PMC9984535; doi:10.1038/s41467-023-36847-9)
Supplement: Supplementary file 3 — Reporting Summary [file 41467_2023_36847_MOESM3_ESM.pdf]

nature portfolio

Corresponding author(s): Fredrik J. Swartling, PhD

Last updated by author(s): Feb 9, 2023

Reporting Summary

Nature Portfolio wishes to improve the reproducibility of the work that we publish. This form provides structure for consistency and transparency in reporting. For further information on Nature Portfolio policies, see our Editorial Policies and the Editorial Policy Checklist.

Statistics

For all statistical analyses, confirm that the following items are present in the figure legend, table legend, main text, or Methods section.

n/a	Confirmed	
☐	☒	The exact sample size (*n*) for each experimental group/condition, given as a discrete number and unit of measurement
☐	☒	A statement on whether measurements were taken from distinct samples or whether the same sample was measured repeatedly
☐	☒	The statistical test(s) used AND whether they are one- or two-sided<br>*Only common tests should be described solely by name; describe more complex techniques in the Methods section.*
☐	☒	A description of all covariates tested
☐	☒	A description of any assumptions or corrections, such as tests of normality and adjustment for multiple comparisons
☐	☒	A full description of the statistical parameters including central tendency (e.g. means) or other basic estimates (e.g. regression coefficient) AND variation (e.g. standard deviation) or associated estimates of uncertainty (e.g. confidence intervals)
☐	☒	For null hypothesis testing, the test statistic (e.g. *F*, *t*, *r*) with confidence intervals, effect sizes, degrees of freedom and *P* value noted<br>*Give P values as exact values whenever suitable.*
☒	☐	For Bayesian analysis, information on the choice of priors and Markov chain Monte Carlo settings
☐	☒	For hierarchical and complex designs, identification of the appropriate level for tests and full reporting of outcomes
☒	☐	Estimates of effect sizes (e.g. Cohen's *d*, Pearson's *r*), indicating how they were calculated

Our web collection on statistics for biologists contains articles on many of the points above.

Software and code

Policy information about availability of computer code

Data collection	RNA-seq: Raw RNA-seq reads were mapped to the mm9 mouse genome via the STAR alignment algorithm (Version 2.7.2b) and the BOWTIE2 alignment algorithm (Version 2.3.4.3). Gene specific read counts were obtained via the featureCounts function from the Subread package (version 1.5.2).<br>Exome Seq: Mouse tumor biopsies, normal control sample pairs and GMYC1 and GTML2 tumor cell lines were whole exome sequenced by the National Genomics Infrastructure SNP&SEQ Technology Platform (Uppsala). Sequencing library preparation were performed using Twist Mouse Exome Panel Kit (Twist Bioscience). Clustering generation and paired-end sequencing were run for 100 cycles in one flowcell using the NovaSeq 6000 system (Illumina). A detailed analysis of specific mutations and allele frequencies on the Trp53 transcript (ENSMUST00000108658.9) on Chr.11 was performed.<br>Methylation Arrays: The methylation in DNA from mouse tumor samples was profiled using the MM285 Infinium MouseMethylation BeadChip (Illumina).
Data analysis	RNA-seq: Differential expression analyses were conducted in R using the edgeR (Version 3.28.1) package. Batch-effect removal was performed via the limma (Version 3.42.2) package. Mouse to Human Ortholog mappings/translation was conducted through the biomaRt (Version 2.42.1) package. The metagene R scripts (Tamayo et al. (2007); https://doi.org/10.1073/pnas.0701068104) was used for cross-species analyses.<br>Exome Seq: Reads were aligned to the GRCm38.p6 reference genome build using Burrows-Wheeler Aligner version 0.7.17. Bam files were converted using Samtools v. 1.14 and duplicated reads where marked using Picard v. 2.23.4 (https://broadinstitute.github.io/picard/). Variants in tumor-normal and tumor-only samples were called with VarScan v. 2.3.9 using somatic and mpileup2cnettings respectively. Somatic variants from tumor-only samples were obtained by excluding all variants reported in the normal samples. All samples were annotated using SnpEff v. 5.0C.<br>Methylation Arrays: Pre-processing of beta values from human samples were performed using the minfi package (v1.24.0) and the IlluminaHumanMethylation450kmanifest (v0.4.0) package. Differential methylation analyses, including significance testing, of CDKN2A specific

CpG probes between MB samples were performed using the dmpFinder function from the minfi package.
Following raw data generation of mouse samples, all IDAT files were processed in R (v4.1.2) using the package SeSAMe (version 1.14.2). and annotated with the MM285 Infinium Mouse Methylation Manifest 12v1-0 manifest. Using the manifest, probes known to be poor quality (e.g., cross-hybridizing, SNP-enriched) were masked and remaining data values normalized using normal-exponential out-of-band (noob) method (Triche et al. (2013); https://doi.org/10.1093/nar/gkt090).
Animal survival was graphically shown as a Kaplan-Meier curve, made and assessed using GraphPad Prism 8 or 9 software.

For manuscripts utilizing custom algorithms or software that are central to the research but not yet described in published literature, software must be made available to editors and reviewers. We strongly encourage code deposition in a community repository (e.g. GitHub). See the Nature Portfolio guidelines for submitting code & software for further information.

Data

Policy information about availability of data

All manuscripts must include a data availability statement. This statement should provide the following information, where applicable:

- Accession codes, unique identifiers, or web links for publicly available datasets
- A description of any restrictions on data availability
- For clinical datasets or third party data, please ensure that the statement adheres to our policy

Raw and processed RNA-seq data for the GMYC and GTML tumor samples have been deposited in the Gene Expression Omnibus (GEO) database and are available via the accession number GSE139240 (https://www.ncbi.nlm.nih.gov/geo/query/acc.cgi?acc=GSE139240). Previously published data used in this study are available from GEO using accession numbers GSE85217 (https://www.ncbi.nlm.nih.gov/geo/query/acc.cgi?acc=GSE85217) and GSE8521232 (https://www.ncbi.nlm.nih.gov/geo/query/acc.cgi?acc=GSE85212), GSE7303844,75 (https://www.ncbi.nlm.nih.gov/geo/query/acc.cgi?acc=GSE73038), GSE12481429 (https://www.ncbi.nlm.nih.gov/geo/query/acc.cgi?acc=GSE124814), GSE162080 31 (https://www.ncbi.nlm.nih.gov/geo/query/acc.cgi?acc=GSE162080) and from Children's Brain Tumor Network76 (https://cbtn.org/). All data supporting the findings of this study are available within the article and its supplementary information files. Source data are provided with this paper.

Human research participants

Policy information about studies involving human research participants and Sex and Gender in Research.

Reporting on sex and gender	The study is not involving human research participants.
Population characteristics	Population characteristics from publically available data mostly includes primary biopsies from tumors in children (under 18 years old) with no selection on a specific sex.
Recruitment	No participants were recruited to this study. We only used publically available data on patient collections where a specific brain tumor was identified upon diagnosis.
Ethics oversight	We used publically available data on patient collections where a specific brain tumor was identified.

Note that full information on the approval of the study protocol must also be provided in the manuscript.

Field-specific reporting

Please select the one below that is the best fit for your research. If you are not sure, read the appropriate sections before making your selection.

☒ Life sciences ☐ Behavioural & social sciences ☐ Ecological, evolutionary & environmental sciences

For a reference copy of the document with all sections, see nature.com/documents/nr-reporting-summary-flat.pdf

Life sciences study design

All studies must disclose on these points even when the disclosure is negative.

Sample size	Sample sizes for in vivo experiments, e.g. treatment cohorts, were used on the basis of agreeing to a size suitable for ethical regulations while also large enough to ensure strong statistical power. Sample sizes were large enough to ensure results obtained were of a representable quantity and quality. For global expression analysis more than 3 samples of each group was usually included.<br>For in vitro experiments, initial samples size for the number of new cell lines generated (GMYC model) was n=3. We also used at least three different lines for dox treatments, DNMT inhibition or HSP90 inhibition. When direct comparisons were used for more precise follow up comparisons - a representative cell line could be directly compared against another representative line. Such experiments were always confirmed and repeated at least three times. When e.g. protein measurements or histological analyses, numerous biological repeats were conducted to ensure a representative overview.
Data exclusions	A SHH MB was excluded from the GTML tumors collected (from GSE162080) and analyzed as it was not designated to the correct human subgroup (Group 3 MB) based on global expression profiling. The rationale for this exclusion has also been described in the manuscript text. For survival analysis of animals that were found dead without obvious signs of tumor (primary tumor penetrance studies) they were marked as censored as described in Methods.

	Finally, to avoid irrelevant survival effects caused by secondary tumors that might arise in long-term survivors, patients with survival times longer than 10 years (according to publically available clinical data) were censored at exactly 10 years as described in Methods. No other data has been excluded from this study.
Replication	For applicable situations, experiments were replicated numerous times (>3). Analysis of animal tissue was carried out for many animals across multiple generations to ensure no genetic changes occured and that all data shown was an accurate and average representation of these samples. Where appropriate, statistical analysis has been carried out for these replicates. The work we present is reliable as it is a combination of many findings, both confirmed in vivo and in vitro. For ethical reasons some animal experiments were not repeated. Instead enough animals were analyzed in order to prove statistically significant differences.
Randomization	Before graft injections, mice injected were randomized into different treatment arms/cages. Animal treatment groups were not randomised. Genotyping was carried out to ensure all mice contained the correct transgenes (GMYC: Glt1-tTA, TRE-MYC and GMYC/ARF model: Glt-tTA-TRE-MYC-ARF fl/fl). Once all mice were confirmed to contain the correct transgenes, cohorts were divided without randomisation. All experiments involving intracranial injections used the same strict injection coordinates and numbers of cells injected, as all injections were carried out by one researcher, to eliminate any bias.
Blinding	Blinding was performed where the responsble researcher blinded results that were analysed/quantified without knowing the name of the investigated sample. In vivo work and mouse symptoms were assessed by multiple researchers as well as the technical staff in the animal facility. Unless stated otherwise, all mice were sacrificed at a humane endpoint judged to be similar across all individual animals.

Reporting for specific materials, systems and methods

We require information from authors about some types of materials, experimental systems and methods used in many studies. Here, indicate whether each material, system or method listed is relevant to your study. If you are not sure if a list item applies to your research, read the appropriate section before selecting a response.

Materials & experimental systems

n/a	Involved in the study
☐	☒ Antibodies
☐	☒ Eukaryotic cell lines
☒	☐ Palaeontology and archaeology
☐	☒ Animals and other organisms
☒	☐ Clinical data
☒	☐ Dual use research of concern

Methods

n/a	Involved in the study
☒	☐ ChIP-seq
☒	☐ Flow cytometry
☒	☐ MRI-based neuroimaging

Antibodies

Antibodies used	β-tubulin (Cell Signalling Technology, Cat # 2146 (1:1000)), Cleaved Caspase-3 [Asp175] (Abcam, Cat # ab49899 (WB 1:500, IHC 1:1500)), CDKN2A/p19ARF (Abcam, Cat # ab80 (1:1000)), cMYC [Y69] (Abcam, Cat # ab32072 (WB 1:1000, IHC 1:500)), Cyclophilin B (Cell Signalling Technology, Cat # 43603 (1:1000)), GFAP [GA5] (Millipore, Cat # MAB3402 (1:1000)), GFP (Abcam, Cat # ab13970 (1:1000)), HSF1 (Abcam, Cat # ab61382 (1:1000)), HSP70 (Abcam, Cat # ab2787 (1:1000)), HSP90 [EPR16621] (Abcam, Cat # ab203085 (1:1000)), Ki67 [SP6] (Abcam, Cat # ab16667 (1:2000)), n-MYC [Abcam, NCM II 100] (Abcam, Cat # ab16898 (1:250)), NPR3 (Abcam, Cat # ab97389 (1:250)), Olig-2 (Millipore, Cat # AB9610 (1:1000)), OTX2 (R&D Systems, Cat # AF1979 (1:500)), p21 (Abcam, Cat # ab109199 (1:1000)), Synaptophysin [SY38] (Millipore, Cat # MAB5258 (1:500)), TUJ1 (Covance, Cat # MMS-435P (1:500)), β-actin (Santa Cruz Biotechnology, Cat # sc-47778 (1:1000)), CDKN2A/p16INK4A (Abcam, Cat # ab211542 (1:1000)), anti-mouse IgG secondary HRP (VWR, Cat # NXA931 (1:1000)), anti-rabbit IgG secondary HRP (GE Healthcare, Cat # NA934 (1:1000)) and Lamin B1 (Abcam, Cat # ab16048 (1:1000)).
Validation	Antibodies were used as per the manufacturers' instructions. For important stainings, antibodies to discover the same protein a comparative analysis was performed.

Eukaryotic cell lines

Policy information about cell lines and Sex and Gender in Research

Cell line source(s)	GMYC1, GMYC2, GMYC3 cell lines were derived from tumors of the transgenic mice generated in this study. GTML2 were derived from a transgenic mouse derived in previously published studies (Swartling et al., Genes & Dev, 2010). GMYC1/+MYC and GTML2/+MYC cell lines were generated from the GMYC1 and GTML2 cell lines previously mentioned, using lentivirally overexpressing MYC, carried out in this study.
Authentication	None of the mouse cell lines used were authenticated as they were directly maintained in low passage numbers after generation. Human lines were analyzed with STR profiling by Sigma Aldrich and ATCC (before ordering from them), to confirm correct donor our species.
Mycoplasma contamination	Cell lines used were contamination-free when obtained from repositories or vendors but tested regularly upon culturing and passaging using a Mycoplasma detection kit (MycoAlert) from Lonza. Only confirmed mycoplasma-free lines were used in research.

Commonly misidentified lines (See ICLAC register)	*Name any commonly misidentified cell lines used in the study and provide a rationale for their use.*

Animals and other research organisms

Policy information about studies involving animals; ARRIVE guidelines recommended for reporting animal research, and Sex and Gender in Research

Laboratory animals	Animal strains involved in this study include a transgenic mouse derived in a previously published study (Swartling et al, Genes & Dev, 2010) and a new transgenic mouse strain crossed from the aforementioned Glt1-tTA mice and a TRE-MYC strain (JAX stock #019376). Further strain crossing with pTRE-H2BGFP (JAX stock #005104), LC1 (JAX stock #006234), ARF (JAX stock #023323), R26R-Confetti (JAX stock #013731).
Wild animals	The study did not involve wild animals.
Reporting on sex	In transgenic animals both female and male mice develop tumors spontaneously (no significant differences). However, animals in where cells were orthotopically transplanted were mostly female due to risk of more fighting in male colonies. Still, here tumor cells are compared with or without treatment, so the sex of the host is considered less important.
Field-collected samples	The study did not involve samples collected from the field.
Ethics oversight	All experiments were performed in accordance with national Swedish guidelines and regulations, and with the approval (C105/16, 5.8.18-16350/2017 and 5.8.18-18303/2021) of the animal care and use committee at Uppsala University, Uppsala, Sweden.

Note that full information on the approval of the study protocol must also be provided in the manuscript.
